# Undergraduate Vaccine Hesitancy and the Influence of “Home” Locales

**DOI:** 10.3390/ijerph192114370

**Published:** 2022-11-03

**Authors:** Tyler W. Myroniuk, Enid Schatz, Michelle Teti, Joan M. Hermsen

**Affiliations:** 1 Department of Public Health, University of Missouri, Columbia, MO 65211, USA; 2 Department of Sociology, University of Missouri, Columbia, MO 65211, USA

**Keywords:** COVID-19, influenza, population health, college students, vaccines

## Abstract

Purpose: In the US, the COVID-19 pandemic has exposed deeply rooted resistance to public health. This has important consequences for SARS-CoV-2 variant spread and for future uptake of influenza and other vaccines. We examine these phenomena in Missouri, where its low vaccination rates, high levels of uninsured residents, predominance of conservative values, and stark rural-urban divides are intricately connected to public health resistance. The Socio-Ecological model guides our approach. Methods: We use data from the Fall 2020 Midwestern University (MWU—a pseudonym) Study of Seropositivity and Risk for SARS-CoV-2 and COVID-19 which are harmonized with the American Community Survey, Missouri County-Level Study, and the USDA Rural-Urban Continuum Codes to estimate multi-level regression models on the relationship between undergraduate students’ “home” locales and their own COVID-19 and influenza vaccine hesitancy. Findings: The availability of primary care physicians, the prevalence of influenza vaccinations, and location type at the county level, as well as the percentage of residents without health insurance at the zip code level differentially predict COVID-19 and influenza vaccine hesitancy. Conclusions: There is a link between county-level health and geographic characteristics, and individuals’—who were influenced by those counties because that is where their parents live—hesitancy towards vaccines. Identifying feasible, precise, local solutions to reducing vaccine hesitancy could take place if community members and other stakeholders would be open to changes in local-level health policy or practice.

## 1. Background

The COVID-19 pandemic has exposed deeply rooted resistance to public health best practices resulting in geographic pockets of vaccine hesitancy in the US. This has important consequences for SARS-CoV-2 variant spread now, but also in future uptake of influenza and other vaccines in order to avoid multiple infectious disease and virus outbreaks from simultaneously occurring and crippling health care systems in the future. Missouri is a microcosm of these uniquely American phenomena as its age structure, low rates of health insurance, predominance of conservative values, and stark rural-urban divides are intricately connected to these issues [1,2,3]. Further, only one Missouri county had at least 50% of its population fully vaccinated by early September 2021, a figure still well below what is needed to reach “herd” or community immunity; by September 2022, only 58% of Missouri’s total estimated 6.1 million residents were fully vaccinated, ranking 11th lowest out of 50 states [4,5,6,7]. Thus, examining these factors in Missouri can offer important insight for ongoing broader American health outreach efforts and messaging ideas—it may be a bellwether for public health, much like Kansas was identified for voting patterns [8,9].

The Socio-Ecological Model is ideal for assessing how factors beyond individual characteristics are linked to individuals’ decisions in the COVID-19 pandemic, including vaccinations [10,11,12,13,14]. While the specific environmental characteristics of the Socio-Ecological model are not the same across different contexts, the model is widely applicable. This model accounts for the reasons—or reasonable explanations—why individuals acquire health beliefs and practices, and how individual, interpersonal, community, institutional, and geographic characteristics are nested and differentially associated with health decisions [15,16,17,18]. For instance, family members, health care providers, and university organizations differentially influence college students’ decisions to get vaccinated for Human papillomavirus (HPV) compared to influenza; at the same time there could be many other—sometimes difficult-to-measure—characteristics such as local politics or cultural attitudes towards medicine also influencing such health decisions [19,20]. Lack of health insurance, politicization of vaccinations, educational achievement, and internet availability all factor into COVID-19 vaccine hesitancy but not uniformly in the same directions due to marked variation in local contexts [14,21,22]. A lack of health insurance or medical facilities in a geographic space could detract individuals from getting vaccinated, or push them to get vaccinated given that the COVID-19 vaccines are free and important for living a less-restricted lifestyle; important to include contextually appropriate measures in estimating effects within a Socio-Ecological frame to minimize error [23,24]. Effectively assessing the micro-, meso-, and macro-level characteristics contributing to a health outcome, such as vaccine hesitancy, requires reliable measurement at each level.

The spatial and geographic variation in Missouri—from the populated metropolitan areas of Kansas City, St. Louis, and Springfield, to isolated urban areas such as Columbia and Joplin, to remote towns in the Ozark Plateau and the Northern Plains—matches well with urban–rural continuum differences pertaining to COVID-19 [25,26,27]. These areas have varied access to services and medical professionals, exposure to COVID-19, and political viewpoints. However, COVID-19 vaccination rates show that remote and conservative regions in Missouri do not always have high vaccine hesitancy, making it important to explore what other factors may be influencing health attitudes and behaviors [4].

Shifting political and financial landscapes have influenced access to care in Missouri. Prior to a 2021 Missouri Supreme Court decision, the Missouri legislature declined the Medicare supplement/expansion, despite a 2020 referendum accepting it [28]. In addition, since 2014, 10 rural hospitals—including 9 acute care hospitals—in Missouri have closed due to financial pressure reducing access to doctors and care in general [29]. All of this underscores the importance of measuring how community and spatial factors are linked to individuals’ public health intentions and actions surrounding infectious diseases and viruses.

Psychological studies and assessments of health care workers have identified a link between prior influenza behavior and perceptions on COVID-19 vaccinations [30,31,32,33,34]. However, to our knowledge, there are no Socio-Ecological Model studies examining both COVID-19 and influenza vaccine hesitancy even though there is one study that descriptively assesses the prevalence of hesitancy for both vaccines among US college students [35]. When considering that the COVID-19 vaccinations are likely to become an annual public health effort, like influenza vaccinations, per US COVID-19 officials Drs. Anthony Fauci and Ashish Jha, and that individuals’ characteristics and environmental context matter, it is important to gauge whether hesitancy is different towards each vaccine [36].

Our study sample consists of college undergraduate students, who have been emblematic of the contradictions regarding public health behavior amid a global pandemic—making them an ideal sub-group to understand. Despite being exposed to scientific principles via higher education, at the beginning of the COVID-19 pandemic, American undergraduate students flaunted public health guidance in large gatherings and parties, as widely reported in news media. However, other students were also models of prosocial behavior—engaging in best public health practices for the benefit of society [37,38]. So, much of the variation within a similar population is plausibly linked to students’ backgrounds. We hypothesize that zip code and county characteristics—from undergraduate students’ “homes” and permanent addresses where family members also reside—such as higher influenza vaccination, greater levels of health insurance coverage, being from metropolitan areas, and having better access to medical services will be strongly and equally linked to lower chances of COVID-19 and influenza vaccine hesitancy.

## 2. Data

### 2.1. Individual-Level Data

From the middle of September to the middle of December 2020, our research team collected survey data on attitudes, behaviors, and beliefs related to SARS-CoV-2 among students, faculty, and staff at Midwestern University (MWU—a pseudonym) in Missouri. The Study of Seropositivity and Risk for SARS-CoV-2 and COVID-19 (under IRB protocol #2028427) captured detailed data prior to the widespread availability of the COVID-19 vaccine but far enough along in the pandemic for individuals to have become infected and to have developed informed perspectives about the virus and prospective vaccine. A total of 28,217 faculty, staff, and students were recruited via stratified random sampling over the course of the semester, with batches of emails sent out to 950 randomly selected undergraduates, 250 randomly selected graduate/professional students, and 400 randomly selected faculty/staff on a bi-weekly, then, later, weekly basis [39]. Of the entire group recruited, 9.0% (*n* = 2530) participated in at least one study element (note that the 28,217 faculty, staff, and students do not encompass the entire campus population). Most importantly, this included 595 undergraduate students, aged 18–24, with permanent addresses in Missouri, who comprise our analytic sample here. The online survey included measures related to COVID-19 attitudes, preventative behaviors, and beliefs that were immediately relevant to the pandemic in Fall 2020, as well as indicative of future public health issues. To the extent possible, we used questions related to COVID-19 that had been used on other surveys in the public domain in the early months of the pandemic (March–July 2020).

The individual—“level 1”—outcome variables forming the basis of our research are: (1) COVID-19 vaccine hesitancy; and (2) influenza vaccine hesitancy. **COVID-19 vaccine hesitancy** was determined based on responses to the following statement: *If a COVID-19 vaccine becomes available, I will get one*. Students who responded affirmatively, either agreeing or strongly agreeing with the statement were considered not to be COVID-19 vaccine hesitant (=0). Those who disagreed, strongly disagreed, preferred not to answer, or answered that they did not know were deemed COVID-19 vaccine hesitant (=1); by the Fall of 2020, with clinical trials well-underway, anything short of an affirmative answer could only be interpreted as hesitant given the polarizing nature of COVID-19 vaccinations. During the survey period, it was also not yet known how a prospective COVID-19 vaccine would be triaged, with health care workers and older individuals having priority over younger individuals when eventually rolled out to the public; it is unlikely that our measure of COVID-19 vaccine hesitancy in the Fall of 2020 would be biased by this eventuality compared to if it were collected in the Spring of 2021. **Influenza vaccine hesitancy** was determined based on two questions about the 2019 and 2020 flu seasons: (a) Did you get a flu shot in 2019 (yes; no)? and (b) Are you going to get a flu shot this Fall (2020) (yes; no; unsure; I already got a flu shot in Fall 2020)? If a student responded that they received their flu shot in 2019 and indicated that they were going to get a flu shot in 2020 or had already received it, they were considered not to be flu vaccine hesitant (=0). If students did not receive a flu shot in 2019, were not planning to receive one in 2020, or were unsure about 2020, they were deemed flu vaccine hesitant (=1). Essentially all individuals in the US (except for those under 6 months old), are eligible for a widely available flu shot each year at no—or low—cost in grocery stores, pharmacies, major events, and in medical clinics; it is a known entity and participants would have a well-established perspective on the choice to opt-out of getting vaccinated.

Other individual-level variables serve as controls in our regression models and are described below. **Racial identity** is dichotomized as those who self-identified as White only (=0) and those who self-identified as Persons of Color—Not White (=1). **Gender identity** is dichotomized as Female (=0) and Male (=1), with 12 cases dropped based on identity outside of this binary. **Age** is a continuous measure of years between date of birth and the date of survey. The **month** when students took the survey ranges from September to December. **Political views** are based on a 7-point scale where participants identified between someone holding “extremely liberal” to “extremely conservative” views, which was re-coded into liberal (=0), moderate (=1), and conservative (=2).

These survey data form the foundation of our analyses; key to our methods are geographic data from undergraduate students: their permanent, “home”, address in Missouri, including zip code and county. These data allow us to harmonize our survey data with public data at various levels of geography.

### 2.2. Zip Code and County-Level Data

To best estimate the extent to which local environmental characteristics predict vaccine hesitancy among undergraduate students, incorporating multiple levels of data is advantageous. Here, data from zip codes—“level 2”—and county—“level 3”—contain relevant health care outcomes in Missouri and allow for enough variation to produce robust estimates; other levels of aggregation, such as the seven regions of the Department of Health & Senior Services offer too little variation while census tracts offer too much to effectively model. Thus, the regression models are not over-specified and the measures—noted below—are conceptually appropriate. Undergraduate students lived in 197 zip codes out of 1024 in Missouri (19.2%) and 65 counties out of 115, including independent cities, in Missouri (56.5%). Six student observations with zip codes that crossed county lines were dropped.

Zip code data come from the American Community Survey (ACS, 2015–2019) on the percentage of **working age adults** (18 to 64 years old), and those **65 years and older** that **do not have health insurance**. County data come from the Missouri County-Level Study (CLS, 2016) on **presence of primary care physicians per 100,000** population and the **percentage of population who received the influenza vaccination**; and US Department of Agriculture (USDA, 2013) **rural-urban continuum classification** codes, with counties dichotomized as metropolitan (=0) and non-metropolitan (=1). Table 1, below, shows the variables in levels 1, 2, and 3. These variables were chosen as those recently available for each zip code and county in Missouri and as the most relevant to health care behaviors and outcomes. Barriers to health care such as lack of insurance and lack of access to physicians, prior mainstream vaccination behavior (influenza), and being in an urban area—also linked to medical facility access—are among the most salient characteristics of geographic spaces pertaining to public health and individuals’ decisions within a health ecosystem [13,14,20]. We examined median zip code and county income levels but do not include these in the models presented here due to issues of collinearity and subsequent model error (see Supplementary Materials for more details).

### 2.3. Analytic Techniques

Multi-level regression modelling techniques are employed to examine relationships between social context and individuals’ attitudes and behaviors. Equation (1), below, shows an example logistic regression estimation procedure.

(1) $$\ln\left(\frac{p_{ijk}}{1-p_{ijk}}\right)=\beta_{0}+\beta_{1}COUNTYVARS_{k}+\beta_{2}ZIPCODEVARS_{j}+\beta_{3}STUDENTVARS_{i}$$

Here, the log odds of an undergraduate (*i*), in zip code (*j*) and county (*k*), in the Fall of 2020, expressing hesitation about hypothetically receiving a COVID-19 and influenza vaccine is predicted by each level of variables. However, the higher-level variables do not each have an intercept that randomly varies; thus, zip code and county variable values are assigned to each student, making them appear as individual-level data. To correct for potential bias in this approach, we account for the clustering of standard errors at the highest level of geography—the county—rather than the individual or zip code [40,41]. Sensitivity analyses with regressions modelling random intercepts at each level of analysis—levels 1 through 3 for instance—do not substantively differ (results not presented but available on request). Additional sensitivity testing indicated that the addition of level 2 variables and level 3 variables incrementally add more explanatory power to our models (via Pseudo R^2^ values roughly equating to variance as found in ordinary least squares regression models).

Further, we test whether students’ home residence is in a non-metropolitan county and students’ political views matter above and beyond other individual, zip code and county effects; to do so we include stepwise models (models 2 and 3 in each table) with these variables.

Finally, we report missing values of each variable in Table 2, below. County- and zip code-level predictors did not have any missing values and, with the exception of political views (7.1%), individual-level variable missingness was negligible. Unfortunately, multiple imputation of political views would be inappropriate given that the majority of missing cases stemmed from “Don’t Know” and “No Answer” values being deemed as such; considering the politicized environment in the US in the Fall of 2020, ahead of a Presidential election, individuals who did not know their political views or would not answer were not a random group—a precondition for multiple imputation. Still, complete case regressions (same N across models) were conducted for sensitivity analyses (not presented) and there were no substantive differences compared to the regressions we present here (different Ns across models).

## 3. Results

### 3.1. Descriptive Statistics

Table 2, above, shows descriptive statistics for individuals. Thus, each respondent has values from the Study of Seropositivity and Risk for SARS-CoV-2 and COVID-19 as well as values for each zip code and county variable.

Notably, a higher proportion of students expressed hesitancy towards the influenza vaccine (42.0%) than a prospective COVID-19 vaccine (0.20). Still, half of these undergraduate students—who have home addresses in Missouri—expressed hesitancy for at least one of two vaccines (results not presented).

Table 3 describes zip code and county predictors at each of their levels of aggregations; this provides a different portrayal of the geographic distribution of outcomes than in Table 2 because students’ home residences are not equally distributed among zip codes and counties. Across Missouri’s counties, there are roughly 50 primary care physicians per 100,000 residents; counties average about 58% of their population receiving the influenza vaccine; and, just over half (56.1%) of study counties are non-metropolitan. Across Missouri’s zip codes, almost 12% of their working-age residents (18 to 64 years old) do not have health insurance; there is almost universal coverage among those 65 years and older, likely due to Medicare availability upon retirement.

### 3.2. Multi-Level Regressions

Our first set of regressions, predicting COVID-19 vaccine hesitancy, are found in Table 4. Across all three models, the more primary care physicians per 100,000 residents in a county is associated with the lower likelihood of being hesitant to a prospective COVID-19 vaccine—when surveyed in the Fall of 2020 (*p* < 0.05 models 1–3). In model 2, the non-metropolitan county dummy variable is introduced, stepwise, but does not add substantive explanatory power—based on goodness of fit tests—and is not statistically significant. In models 1 and 2, which do not control for political views, there is a positive association between the higher the percentage of 18- to 64-year-old residents without health insurance and undergraduate student COVID-19 hesitancy (*p* < 0.05 model 1; *p* < 0.10 model 2); the inclusion of students’ political views (in models 3) nullifies that effect but not the coefficient for primary care physicians per 100,000 residents.

Our second set of regressions, predicting influenza vaccine hesitancy, are found in Table 5. Unlike in Table 4, there is no association between the number of primary care physicians per 100,000 residents and influenza vaccine hesitancy. However, across all models, there is an unexpected relationship where higher the percentage of the county who received an influenza vaccine, the higher the chances of an undergraduate being hesitant about receiving it (*p* < 0.05 model 1; *p* < 0.01 models 2 and 3). Additionally, there is a positive association between non-metropolitan counties and influenza vaccine hesitancy (*p* < 0.05 models 2 and 3); this indicator adds a consequential amount of explanatory power beyond model 1, based on the pseudo R^2^ and AIC values. Health insurance at the zip code level is not associated with influenza vaccine hesitancy. Lastly, the inclusion of undergraduate students’ political views appears to amplify the non-metropolitan county effects and accounts for a large amount of model explanatory power.

## 4. Discussion

County characteristics were most salient in predicting students’ vaccine hesitancy. The presence of primary care physicians in counties is most strongly and consistently associated with lower chances of being COVID-19 vaccine hesitant. However, it is unclear how the presence of primary care physicians could affect COVID-19 vaccine hesitancy or if it is a proxy for counties that have a higher demand for primary care—suggesting that its population is enthusiastic about health care (which there are currently no population-level estimates of); future research needs to address whether the mere presence of more physicians or placing physicians in key places to ease access—especially in underserved areas—would affect this relationship. The same goes for counties that have higher levels of working-age adults without health insurance; undergraduates’ vaccine hesitancy could be a by-product of larger health-resistant forces, as distrust in medicine or science, that manifest in lower COVID-19 vaccination rates (which there are also currently no population-level estimates of). Furthermore, notably, being from a non-metropolitan county is associated with higher chances of being influenza vaccine hesitant. This is further complicated by the amplification of influenza vaccine hesitancy in non-metropolitan counties when political views are factored in; the marked individual conservative effects, relative to being liberal, affirm the presumed link between politics and health decisions. In short, COVID-19 and influenza vaccine hesitancy are different from one another and part of this could be due college students being unsure about the safety or efficacy of the COVID-19 vaccine—since it was a new vaccine—as was found in a similar college town setting [42]. Qualitative methods would be better suited to uncovering these mechanisms in subsequent research.

Understanding why they are different should be a next consequential step in scholarship, given that the COVID-19 vaccine could become necessary on an annual basis as is the influenza vaccine. Health communication and health advocacy may be more visible in counties with more health care infrastructure, and this may be important when new viruses emerge, and people are still wary about exposure. Identifying the appropriate messaging will be crucial.

Undergraduate students are a product of their homes and are shaped by the colleges they attend. Consequently, they offer insight into the complex dynamics of American public health efforts. Although our sample is not representative of students in Missouri, let alone across all states, there are parallels in other states that ought to be explored—especially for students at universities in states with considerable geographic variation in access to health care, political views, and economic opportunities such as Arkansas, Kentucky, Michigan, Mississippi, Oklahoma, Pennsylvania, and Wisconsin. Evidence from a rural Pennsylvania college town indicates that adult residents—not college students—showed remarkable lack of trust in health systems which led to COVID-19 vaccine hesitancy; the surrounding rural context and associated conditions, like ours, are linked to vaccine hesitancy [43].

While our analyses only examine associations, and are not causal, the relationship between the characteristics of MWU undergraduates’ “home” residences and their intentions to get vaccinated for COVID-19 and influenza, suggest that student health decision making is more complicated than simply being exposed to campus messaging and best public health principles promoted by the university community. These students therefore must be factoring in various types of information when deciding about getting vaccinated and that different information is prioritized based on where students home residences are located.

## 5. Conclusions

Undergraduate students are, arguably, among the largest sub-populations in America to be consistently exposed to scientific principles; if there were a sub-population that would comply with vaccinations, undergraduate students would be one. Even if there is a risk of the ecological fallacy, the findings that zip code and county-level characteristics are associated with vaccine hesitancy among undergraduates in Missouri implies that local context could have a wider influence on its general population.

What this all means for the continued fight against COVID-19 transmission, seasonal influenza, and unforeseen future pandemics remains to be seen. A burgeoning population precision health field, within the larger precision health movement, suggests that targeted, highly tailored messages to sub-populations would be necessary to reduce vaccine hesitancy—not a “one size fits all” approach. Our research provides a gateway into population precision health by first identifying that there is a link between county-level health and geographic characteristics, and individuals’—who were influenced by those counties because that is where their parents live—hesitancy towards vaccines. The next step is to identify specific counties where these associations are strongest and uncover mechanisms, through qualitative work, as to how local context or even extended family, and intergenerational, pressures might influence individual health decisions. After this, identifying feasible solutions to reducing vaccine hesitancy could take place if community members and other stakeholders would be open to a local-level health policy or practice changes. None of this will happen overnight, but hopefully before the next major public health crisis.

## Figures and Tables

**Table 1:** Analytic Variables by Level and Type.

	Variable Type
Individual-Level 1	
*COVID-19 Vaccine Hesitancy (vs. not)*	Outcome
*Influenza Vaccine Hesitancy (vs. not)*	Outcome
*Not White (vs. White)*	Control
*Male (vs. Female)*	Control
*Age at Survey (Continuous)*	Control
*Survey Month in 2020*	Control
*Political Views*	Control
**Zip Code Predictors-Level 2**	
*Percentage 18–64 Without Health Insurance*	Predictor
*Percentage 65+ Without Health Insurance*	Predictor
**County Predictors-Level 3**	
*Primary Care Physicians per 100 k*	Predictor
*Percent Who Received Flu Shot*	Predictor
*Non-Metro Counties (vs. metro)*	Predictor

**Table 2:** Individual-Level Descriptive Statistics.

	% or Mean	Standard Deviation	*n*	Missing (%)
**Student Outcomes**				
*COVID-19 Vaccine Hesitancy (vs. not)*	20.4%		588	1.2%
*Influenza Vaccine Hesitancy (vs. not)*	42.0%		574	3.5%
**County Predictors**				
*Primary Care Physicians per 100 k*	80.9	33.9	593	0.0%
*Percent Who Received Flu Shot*	61.4	5.6	595	0.0%
*Non-Metro Counties (vs. metro)*	13.6%		595	0.0%
**Zip Code Predictors**				
*Average Percentage 18–64 Without Health Insurance*	9.5	5.4	595	0.0%
*Average Percentage 65+ Without Health Insurance*	0.3	0.6	595	0.0%
**Student Controls**				
*Not White (vs. White)*	11.6%		594	0.2%
*Male (vs. Female)*	30.5%		583	2.0%
*Age at Survey (Continuous)*	20.4	1.4	595	0.0%
*Survey Month in 2020*			595	0.0%
September	10.9%			
October	31.6%			
November	44.2%			
December	13.3%			
*Political Views*			553	7.1%
Liberal	55.2%			
Moderate	15.6%			
Conservative	29.3%			

Note: Means do not have percentage symbols next to their values in the second column.

**Table 3:** County and Zip Code-Level Descriptive Statistics.

	% or Mean	Standard Deviation	*n*
**County Predictors**			
*Primary Care Physicians per 100 k*	50.0	28.2	66
*Average Percent Who Received Flu Shot*	58.5	6.1	66
*Non-Metro Counties (vs. metro)*	56.1%		66
**Zip Code Predictors**			
*Average Percentage 18–64 Without Health Insurance*	11.6	6.4	198
*Average Percentage 65+ Without Health Insurance*	0.4	0.9	198

Note: Means do not have percentage symbols next to their values in the second column.

**Table 4:** Multi-Level Logistic Regressions Predicting the Log Odds of COVID-19 Vaccine Hesitancy among Undergraduates.

	(1)	(2)	(3)
**Level 3-County** (N_max_ = 65)			
*Primary Care Physicians per 100 k*	−0.01 *	−0.01 *	−0.01 *
*Percent Who Received Influenza Vaccine*	−0.00	−0.00	−0.00
*Non-Metro Counties (ref. metro)*		−0.03	−0.01
**Level 2-Zip Codes** (N_max_ = 197)			
*Percentage 18–64 Without Health Insurance*	0.03 *	0.04 ^†^	0.03
*Percentage 65+ Without Health Insurance*	−0.19	−0.19	−0.33
**Level 1-Students** (N_max_ = 595)			
*Not White (ref. White)*	0.26	0.25	0.57
*Male (ref. Female)*	−0.28	−0.28	−0.55 **
*Age at Survey (Continuous)*	0.12	0.12	0.20 *
*Survey Month in 2020 (ref. September)*			
October	0.30	0.30	0.30
November	0.00	0.00	0.08
December	0.56	0.56	0.54
*Political Views (ref. Liberal)*			
Moderate			0.75 ***
Conservative			1.20 ***
*Constant*	−3.65 *	−3.64 *	−5.80 **
Observations	573	573	540
Pseudo *R*^2^	0.031	0.031	0.080
*AIC*	586.78	588.77	525.17
*BIC*	634.64	640.98	585.25

^†^*p* < 0.10, * *p* < 0.05, ** *p* < 0.01, *** *p* < 0.001.

**Table 5:** Multi-Level Logistic Regressions Predicting the Log Odds of Influenza Vaccine Hesitancy among Undergraduates.

	(1)	(2)	(3)
**Level 3-County** (N_max_ = 65)			
*Primary Care Physicians per 100 k*	−0.00	−0.00	−0.00
*Percent Who Received Influenza Vaccine*	0.04 *	0.04 **	0.04 **
*Non-Metro Counties (ref. metro)*		0.80 *	0.86 *
**Level 2-Zip Codes** (N_max_ = 197)			
*Percentage 18–64 Without Health Insurance*	0.04 ^†^	0.02	0.02
*Percentage 65+ Without Health Insurance*	0.02	0.05	−0.07
**Level 1-Students** (N_max_ = 595)			
*Not White (ref. White)*	0.31	0.36	0.48
*Male (ref. Female)*	0.55 ***	0.58 ***	0.52 ***
*Age at Survey (Continuous)*	−0.12 ^†^	−0.13 ^†^	−0.10
*Survey Month in 2020 (ref. September)*			
October	−0.24	−0.28	−0.38
November	−0.31	−0.30	−0.26
December	−0.46	0.50	0.50
*Political Views (ref. Liberal)*			
Moderate			0.48 ^†^
Conservative			0.77 ***
*Constant*	−0.25	−0.58	−1.46
Observations	559	559	522
Pseudo *R*^2^	0.043	0.052	0.075
*AIC*	749.32	744.24	681.25
*BIC*	796.91	796.16	740.86

^†^*p* < 0.10, * *p* < 0.05, ** *p* < 0.01, *** *p* < 0.001.

## Data Availability

The data presented in this study may be available on request from the corresponding author. The data are not publicly available.

## Supplementary Materials

The following supporting information can be downloaded at: https://www.mdpi.com/article/10.3390/ijerph192114370/s1, File S1: The issue of median household incomes for zip codes and counties.
