# Supplementary material for: Undergraduate Vaccine Hesitancy and the Influence of “Home” Locales
Source: Int J Environ Res Public Health. 2022 Nov 3;19(21):14370. doi: 10.3390/ijerph192114370 (PMC9653726; doi:10.3390/ijerph192114370)
Supplement: Supplementary file 1 [file ijerph-19-14370-s001.zip › ijerph-1954330-File S1.pdf]

Supplementary Materials:

The issue of median household incomes for zip codes and counties

There are no significant or substantive bi-variate relationships (via t-tests) between county median household income and COVID-19 vaccine hesitancy nor influenza vaccine hesitancy. Nor is there a significant nor substantive bi-variate relationship between zip code median household income and COVID-19 vaccine hesitancy. However, students who were influenza vaccine hesitant came from zip codes with an average median household income almost $7,000 less than those who were not vaccine hesitant. But, most importantly, county and zip code wealth are too closely correlated with zip code level health insurance for 18–64-year-olds (Pearson's R values of -0.544 and -0.690, respectively) to be appropriately included in the existing models (see Table S1 and S2, below, outlining our sensitivity analyses). We expected county median household income to be closely correlated with the number of physicians per 100,000 people in a county, but these are only weakly, and inconsequentially, correlated (Pearson's R value of -0.068). Consequently, there is a surprising positive, significant association between county median household income and vaccine hesitancy after accounting for other variables found in the full model (model 1, Table S1); it would seem that students from higher income counties are more vaccine hesitant. Yet, further testing finds this to be a function of modelling bias, given the strong correlation with zip code level insurance coverage for 18-64-year-olds; the surprising positive relationship between county median household income and COVID-19 vaccine hesitancy only exists in models where this zip code level health insurance variable is included. This leads us to believe that it would be harmful to formally present such findings in the main text due to their inaccuracies. Inclusion of county median household income

does not substantively affect estimates of influenza vaccine hesitancy and is also excluded from the main text.

Table S1: Multi-Level Logistic Regressions Predicting the Log Odds of COVID-19 Vaccine Hesitancy among Undergraduates—Median Household Income Sensitivity Analyses

	(1)	(2)	(3)
**Level 3-County** (N_{max}=65)			
Primary Care Physicians per 100k	-0.01	-0.01†	EXCLUDED
Percent Who Received Influenza Vaccine	-0.00	-0.01	-0.00
Non-Metro Counties (ref. metro)	0.20	0.28	0.39
COUNTY MEDIAN HOUSEHOLD INCOME ($10k increments)	0.17†	0.42	0.25***
**Level 2-Zip Codes** (N_{max}=197)			
Percentage 18-64 Without Health Insurance	0.05†	EXCLUDED	0.06*
Percentage 65+ Without Health Insurance	-0.37	-0.28	-0.39
Observations	540	540	542

† p<0.10, * $p < 0.05$, ** $p < 0.01$, *** $p < 0.001$. Level 1 variables (race, gender, age, survey month, and political views) and the constant are suppressed for brevity in this table. Median household income estimates come from the American Community Survey (2016-2020 for county estimates, 2015-2019 for zip code estimates).

Table S2: Multi-Level Logistic Regressions Predicting the Log Odds of Influenza Vaccine Hesitancy among Undergraduates—Median Household Income Sensitivity Analyses

	(1)	(2)	(3)
**Level 3-County** (N_{max}=65)			
Primary Care Physicians per 100k	-0.00	-0.00	EXCLUDED
Percent Who Received Influenza Vaccine	0.04**	0.04**	0.04**
Non-Metro Counties (ref. metro)	0.92**	0.06*	0.93**
COUNTY MEDIAN HOUSEHOLD INCOME ($10k increments)	0.04	-0.01	0.51
**Level 2-Zip Codes** (N_{max}=197)			
Percentage 18-64 Without Health Insurance	0.03	EXCLUDED	0.03
Percentage 65+ Without Health Insurance	-0.08	-0.03	-0.09
Observations	522	522	524

† p<0.10, * $p < 0.05$, ** $p < 0.01$, *** $p < 0.001$. Level 1 variables (race, gender, age, survey month, and political views) and the constant are suppressed for brevity in this table. Median household income estimates come from the American Community Survey (2016-2020 for county estimates, 2015-2019 for zip code estimates).
